# ‘No words’—Machine‐learning classified nonverbal immediacy and its role in connecting teacher self‐efficacy with perceived teaching and student interest

**DOI:** 10.1111/bjep.12732

**Published:** 2024-12-29

**Authors:** Rebecca Lazarides, Jonas Frenkel, Uroš Petković, Richard Göllner, Olaf Hellwich

**Affiliations:** ^1^ Department of Educational Sciences University of Potsdam Potsdam Germany; ^2^ Science of Intelligence Research Cluster of Excellence Berlin Germany; ^3^ Technische Universität Berlin Berlin Germany; ^4^ Hector Research Institute of Education Sciences and Psychology University of Tübingen Tübingen Germany

**Keywords:** machine learning, motivation, nonverbal behaviours, student interest, teacher self‐efficacy

## Abstract

**Background:**

Much is known about the positive effects of teachers' self‐efficacy on instruction and student outcomes, but the processes underlying these relations are unknown.

**Aims:**

We aimed to examine the effects of teacher self‐efficacy for student engagement (TSESE) before a lesson on teachers' nonverbal immediacy (NVI) and their enthusiastic teaching. Furthermore, we examined how NVI and enthusiastic teaching affected students' interest after the lesson, controlling for prior interest.

**Sample:**

We used data from the German TALIS video study in the context of the international TALIS study. The study included 50 teachers (46% women) and their 1140 students (53% girls; age*M* = 15 years).

**Methods:**

We developed a computational model to assess teachers' NVI on classroom video data. Using a multimodal longitudinal approach, we tested sequential processes with multilevel path models.

**Results:**

TSESE before the lesson (Time 1) was positively and significantly related to teachers' NVI during the lesson (Time 2). Teachers' NVI (Time 2) was positively related to class‐level enthusiastic teaching behaviours, reported after the lesson (Time 3). Student‐reported enthusiastic teaching behaviours (Time 3) were significantly and positively associated with students' interest (Time 3) when controlling for students' prior interest (Time 1). Students' interest after the lesson (Time 3) was significantly and positively related to students' interest 6 weeks later (Time 4).

**Conclusions:**

Nonverbal behaviours of the teacher are central to classroom instruction by promoting students' perceptions of the teachers' enthusiastic teaching behaviours.

## INTRODUCTION

Teachers' motivations to teach are central to high‐quality instruction and students' academic outcomes (Fives & Buehl, 2016; Lazarides & Schiefele, 2021; Watt & Richardson, 2008). One important component of teacher motivation is teachers' self‐efficacy, that is, their judgement of their own capability to bring about desired outcomes of student engagement and learning, even when students are difficult or unmotivated (Tschannen‐Moran & Woolfolk Hoy, 2001). Although much is known about the positive effects of teacher self‐efficacy on instruction and student outcomes (Kim & Seo, 2018; Klassen & Tze, 2014), it remains to be answered through which processes teacher self‐efficacy is transmitted to students (Lauermann & ten Hagen, 2021). Based on the assumptions of social cognitive theory (Bandura, 1997), in this study, we examine sequential effects from teacher self‐efficacy for student engagement (TSESE) to teachers' in‐class behaviours to students' interest. Concretely, we examine whether TSESE positively affects teachers' nonverbal immediacy (NVI), objectively assessed via machine learning and student‐reported teachers' enthusiastic teaching behaviours, ultimately resulting in increased student interest. Nonverbal immediacy (NVI) can be conceptualized as the entirety of behaviours of individuals that convey positive signals to communication partners, such as sympathy and warmth, thereby reducing the psychological distance between them and fostering a sense of closeness and accessibility (Mehrabian, 1968; Richmond et al., 2003). Teachers' NVI is closely related to enthusiastic teaching behaviours (Keller et al., 2016). Using a multimodal longitudinal methodological approach, we assess teachers' NVI using a computer vision‐based computational model (Petković et al., 2024) and combine it with teacher reports of TSESE and student reports on teachers' enthusiastic teaching behaviours and students' interest.

### Teacher self‐efficacy for student engagement: Relations to instructional processes

Teacher self‐efficacy for student engagement (TSESE) assesses the teachers' own evaluations of their capability to help students value learning as well as to motivate students, particularly those who show low interest in learning (Tschannen‐Moran & Woolfolk Hoy, 2001). Tschannen‐Moran and Woolfolk Hoy (2001) differentiate three dimensions of teacher efficacy—for student engagement, for instructional strategies and for classroom management. These dimensions are independent subdimensions of teachers' general self‐efficacy, and thus, can be seen as a task‐specific assessments that reference a particular area of teaching (Lauermann & ten Hagen, 2021). In our study, we refer to TSESE because this dimension of teacher self‐efficacy refers to the domain of motivating students, and we were interested in motivational processes in class and the prediction of student interest in this study.

TSESE has been shown to positively relate cross‐sectionally to teacher‐reported classroom climate (Fackler et al., 2021) and to positively relate longitudinally both to teacher‐reported relationships with students (Lazarides, Watt, & Richardson, 2023) and to students' class‐level interest via student‐perceived emotional support in class (Lazarides, Schiefele, et al., 2023). However, the processes underlying such relations are unknown. On a theoretical level, it has been proposed that TSE guides teachers' goal direction, their decision‐making and their verbal and nonverbal engagement in aiming to realize their instructional goals (Tschannen‐Moran et al., 1998). Thus, it could be assumed that the link between teachers' TSESE and teaching styles or student outcomes is the teachers' motivating nonverbal and verbal actions (Richmond et al., 2003). However, existing work has not directly addressed the theoretically assumed mechanisms through which teachers' self‐efficacy finds its correspondence on the side of students. Most importantly, the existing approaches may not be sophisticated enough to capture all the nuances of teacher behaviours during a lesson.

### Teacher non‐verbal immediacy, teaching behaviours and student motivation

In the context of education, teacher immediacy encompasses highly situation‐specific verbal and nonverbal behaviours, such as maintaining eye contact with students, using positive facial expressions, employing a relaxed body language or optimizing physical proximity to engage with students (Andersen et al., 1979; Chesebro & McCroskey, 2001). In our study, in accordance with Witt and Wheeless (2001), we define NVI as a composite of the teachers' proximity (approaching students vs. keeping distance), body posture (open, relaxed vs. closed, formal), gestures (using hands and arms vs. stiff, infrequent or ineffective) and facial expression (pleasant, smiling vs. frowning or lack of expression). Meta‐analytical evidence showed on average moderate effect sizes for associations between NVI and students' affective learning (*r* = .48 in Harris & Rosenthal, 2005; *r* = .49 in Witt et al., 2004) and small effects on cognitive performance (*r* = .11 in Harris & Rosenthal, 2005; *r* = .17 in Witt et al., 2004).^1^ A recent literature review (Liu, 2021) also revealed that NVI is a critical factor for learning processes, particularly in the motivational‐affective domain. It is, however, unclear which processes underly the relations between NVI and student outcomes.

We propose that teachers' NVI increases students' interest by fostering their perception of higher levels of enthusiastic teaching behaviours. This assumption is based on a social cognitive perspective on learning and teaching proposing learning as a reciprocal interaction between a person's cognitive and affective processes and behavioural and environmental influences (Bandura, 1986). Thus, the individual's perception of environmental factors, such as the teachers' behaviours, matters for motivational processes of the individual (and vice versa). Accordingly, research has shown that the perspective of students about their teaching are an important intermediary between the actual behaviours of teachers and the actual performance of learning activities by each student (den Brok et al., 2006). Accordingly, we examined how the teachers' behaviours—conceptualized as machine‐learning assessed nonverbal immediacy, affect the students' perceptions of enthusiastic teaching, which in turn was assumed to affect the students' interest in the content taught.

Whereas immediacy refers to teachers' verbal and nonverbal behaviours that enable teachers to increase psychological closeness with students (Mehrabian, 1968), enthusiastic teaching behaviours have been defined as a culmination of expressive behaviours including, for example, vocal delivery, eye contact, demonstrative gestures or body movements that signal an overall high level of energy (Collins, 1978). A decade ago, Keller et al. (2014) distinguished teacher enthusiasm into the perceived inner state of the teacher (‘positive affect’) and the displayed behaviours (‘positive emotional expressivity’) of the teacher. Following theoretical concepts of emotional contagion (Hatfield et al., 1993), teachers convey their emotional states to their students through their behavioural expression as individuals have ‘the tendency to automatically synchronize expressions, vocalizations, postures, and movements with those of another person's’ (page 96). Applying such theoretical assumptions to the context of teaching, our assumption is that teachers use nonverbal social cues in a lesson to communicate, and that students perceive and process such social signals, in turn, influencing their own motivations. Thus, in our study, we focus on lesson‐specific interactions, which occur in specific situations and learning settings and are part of long‐term interactions that may span several weeks, a school year or even multiple school years.

### Aims and research questions

Based on conceptual models of TSE and its effects on teaching performance (Tschannen‐Moran et al., 1998), we assumed that TSE before the lesson (Time 1) would positively and directly relate to teachers' nonverbal immediacy during the lesson (Time 2) (Hypothesis 1).

**FIGURE 1:**
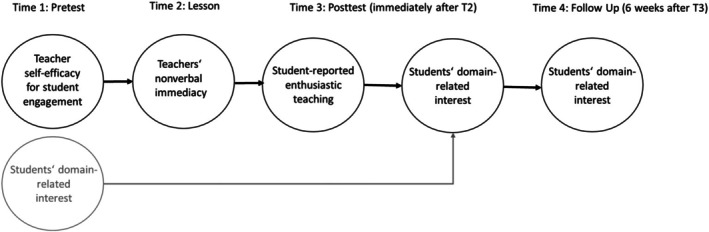
Schematic depiction of the hypothesised model.

In line with the theoretical approaches examining nonverbal immediacy (Mehrabian, 1968; Richmond et al., 2003), we expected that teachers' NVI during the lesson (Time 2) would positively and directly relate to students' perceptions of the teachers' enthusiastic teaching behaviours during the lesson, assessed directly after the lesson (Time 3) (Hypothesis 2). The hypothesised model along with the time sequence of the tested variables is depicted in Figure 1.

Referring to research revealing the positive effects of teachers' enthusiastic teaching behaviours on student motivation (Frenzel et al., 2018), we expected that student‐rated enthusiastic teaching behaviours (Time 3) would be positively associated with students' mathematics interest directly after the lesson (Time 3) when controlling for students' interest before the lesson (Time 1). Additionally, we aimed to examine whether these expected effects would also persist for students' interest 6 weeks later (Time 4) (Hypothesis 3).

We included teachers' years of experience as a covariate because empirical work indicates that teaching experience is significantly and positively related to teachers' self‐efficacy for student engagement (Lazarides, Watt, & Richardson, 2023), and negatively related to teachers' enthusiastic teaching style (Burić et al., 2023).

## METHOD

### Sample

We used data from the German TALIS video study (Grünkorn et al., 2020), which was funded by the Leibniz Association (2017–2020) in the context of the international TALIS study (Teaching and Learning International Survey; OECD, 2020). The study included 50 teachers (46% women; age*M* = 43 years) and their 1140 students (53% girls; age*M* = 15 years) who were distributed across grades 8 (10%), 9 (79%) and 10 (11%). The majority of the 38 participating schools were academic‐track schools (‘Gymnasien’; *n* = 30), followed by comprehensive schools (‘Gesamtschulen’; *n* = 4), secondary schools (‘Realschulen’; *n* = 2), an upper secondary school (‘Oberschule’) and a vocational school (‘Berufsschule’). The TALIS video study focused on mathematics lessons in grades 8–10 in public schools in Germany. We used data from the longitudinal pre‐, post‐ and follow‐up tests of the study. The pretest included standardized surveys for students and teachers and was conducted before the lesson (Time 1). Three lessons selected by the 50 participating teacher were recorded on video (Time 2). The post‐test was conducted immediately after the lesson (Time 3). The follow‐up survey was conducted approximately 6 weeks after the lesson (Time 4).

### Measures

#### NVI model

**FIGURE 2:**
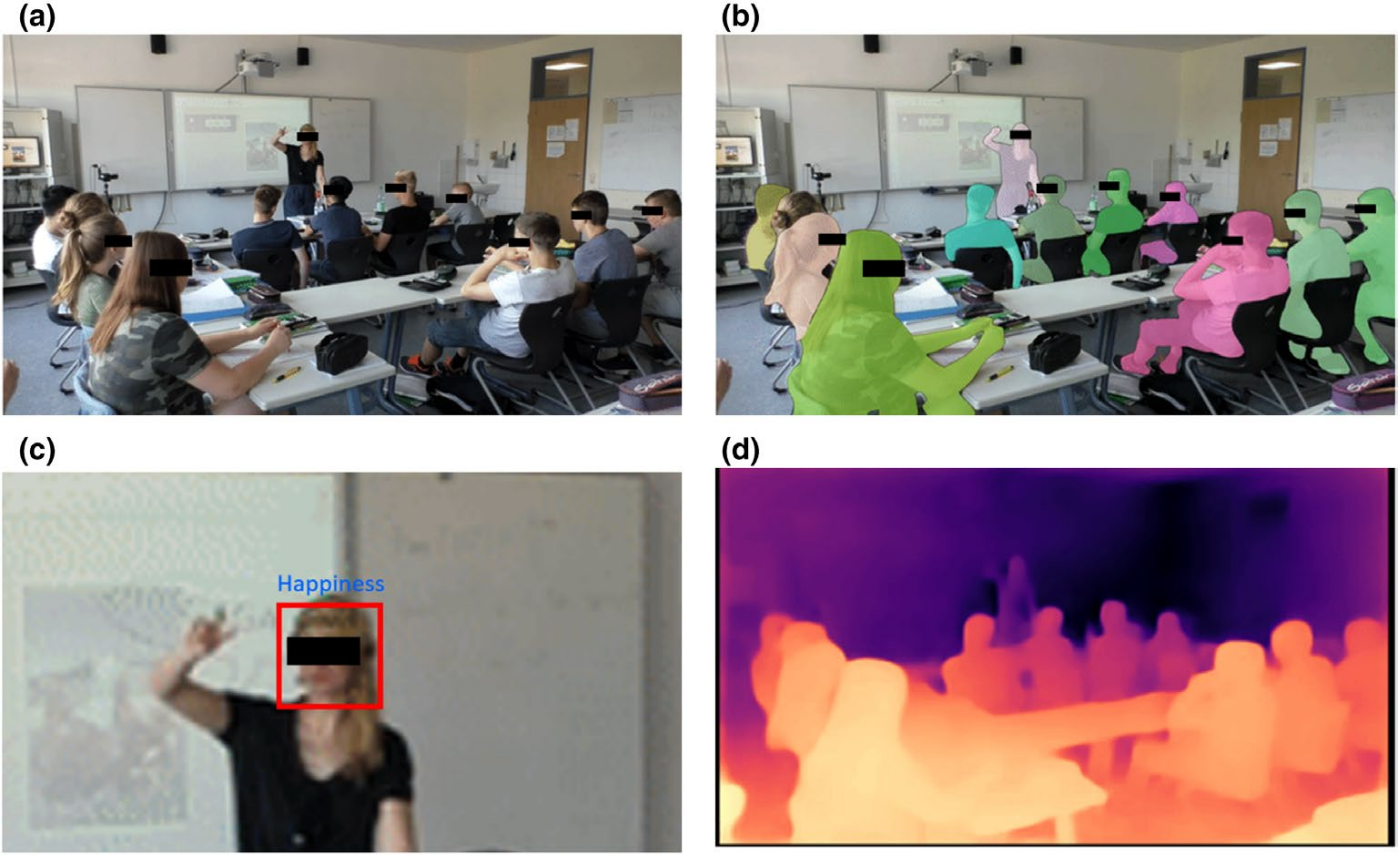
Visualization of the preprocessed video inputs. (a) Red, green, blue (RGB) video input; (b) video segmentation, identification of the teacher and students in the video data; (c) estimation of facial emotional display; (d) generated depth image of the classroom situation.

The NVI of the teachers was assessed using a computer vision‐based machine learning model. Reflecting the multimodal nature of nonverbal social communication, the architecture of the model comprised submodels assessing relevant behavioural dimensions (gesture intensity, perceived physical distance, facial expression), as well as an overarching main model that computationally integrated the individual ratings into an overall NVI score. Our reason for developing a machine learning approach to study teachers' NVI was to make the assessment more automated and efficient. The pipeline summary of the development of the NVI score is depicted in Figure 2 (black rectangles are only included for reasons of anonymization in this manuscript).

From the 50 teachers in the original sample, four classrooms were excluded from the analysis, either because no video material was available or because the video quality was deemed insufficient for computer vision‐based analysis. Consequently, videos from a total of 46 teachers were utilized. For *N* = 43 teachers, three suitable video recordings of one lesson each were available for analysis. For the remaining *n* = 3 teachers, only two videorecorded lessons were available, resulting in a total of 135 videorecorded lessons used for the analysis. The video‐recorded lessons focused on quadratic equations taught to students in grades 9 and 10. In the first step, segments that were suitable for computer vision analysis were identified. Included were segments without excessive camera movement where the teacher delivered frontal presentations, remained fully visible and did not leave the video frame. Following these criteria, a total of 403 30‐s segments were used to train and validate the model.

**FIGURE 3:**
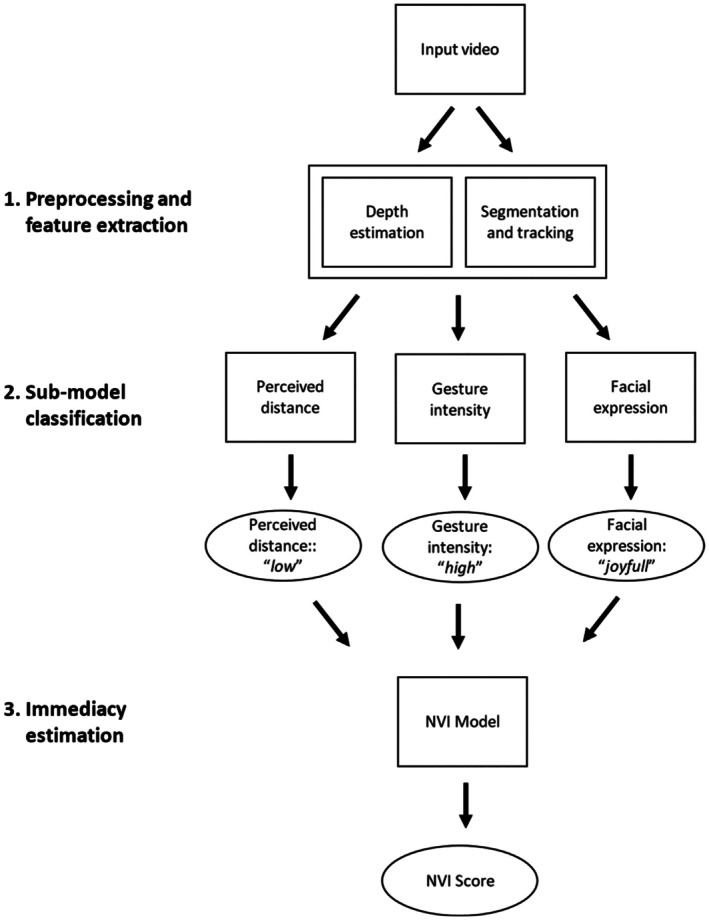
Pipeline summary. The system processes RGB videos to perform tracking, segmentation and depth estimation. Teacher's facial expressions and gesture intensity are estimated from segmentation mask data. Estimates of perceived distance are generated using depth images and segmentation masks of both teachers and students.

In the second step, human raters received an intense training on rating body posture, gesture intensity, perceived physical distance between the teacher and the nearest student and NVI in the videos. A total of three raters were involved in this step and participated in a rater training. The NVI was evaluated based on the unprocessed RGB videos and each video was evaluated by each rater. The raters were students and employees of a German university and were familiar with the typical teaching situations that occur in German educational institutions. The data were labelled by the human raters in terms of these criteria variables. To this end, we randomly selected 3056 frames from the total sample of 403 video segments (302,250 frames). Each of the raters evaluated each of the 3056 frames on a continuous slider scale with regard to body posture, gesture intensity and perceived distance. In addition, raters judged teachers' overall NVI based on the full 30‐s segments using a continuous slider scale. Each of the raters evaluated teachers' NVI on a slider scale for all 400^2^ video segments (the prompt was ‘Please rate the teachers’ nonverbal immediacy). To test the interrater reliability of the human ratings, correlations among the judgements of the three raters were analysed with a two‐way random model (ICC(2,3)). In a two‐way random model, raters are assumed to be randomly selected from a larger population, and each rater rates each individual frame (Shrout & Fleiss, 1979). The rating process revealed a low reliability of ratings for body posture in terms of the teacher's relaxation (ICC(2,3) = .59), even after joint discussions and several rounds of ratings, which is why we excluded this variable from the subsequent analyses. There was an acceptable interrater agreement for distance with an ICC(2,3) of .68 and for NVI with an ICC(2,3) of .68, and a good interrater agreement for gesture intensity, ICC(2,3) of .89 (Cicchetti, 1994). In the third step, the labelled data were used to train and validate the model. For this process, the original 403 video segments for the human rating process were split into a training (324 segments) and a validation (79 segments) data set. Segments lacking sufficient agreement among human raters were excluded from the training data set. Consequently, the following segments/frames were included: gesture intensity and perceived distance, 324 segments (1604–2121 frames); NVI, 316 segments. For the validation data set, all segments were retained. To increase the validity of the model as well as to ensure the generalizability of the NVI assessments, a cross subject (X‐Sub) evaluation protocol was employed, meaning that each teacher was either part of the training data set or part of the validation data set, but never in both. This procedure, commonly used in computer vision for human behaviour analysis (Koelstra et al., 2011; Lin et al., 2020; Shahroudy et al., 2016), is crucial to ensure that the computer vision‐based analysis was actually based on the relevant non‐verbal cues, rather than memorizing individual teachers' behaviours. The division thus allows for assessing NVI in an unseen data set, meaning that it can generalize to new data without prior exposure to the individual teachers. For preprocessing, segmentation masks of the video material were generated to identify the position of both teachers and students. Furthermore, a fine‐tuned ResNet architecture (He et al., 2016) was developed to improve the video quality for the facial expression analysis as well as the gesture intensity and distance models. To allow for reliable distance estimations from 2‐D video data, a depth image was created from the RGB frame (Oquab et al., 2023), enabling the model to recognize spatial relationships between teachers and students. The regressor models for gesture intensity and perceived distance were trained using the human ratings. Additionally, facial expression analysis was performed on the basis of teachers' segmentation masks for the complete sample of 403 segments using the HSEmotion library (Savchenko, 2021). Facial expressions were operationalized via theory‐based dimensions of facially expressed emotional states, including anger, contempt, disgust, fear, happiness, neutrality, sadness and surprise. Finally, the overall NVI model integrated the outputs of the gesture intensity and distance models, as well as the facial expression analysis into a 10‐dimensional vector space, which was then fed into a multilayer perceptron model (Rumelhart et al., 1986) with three fully connected layers. The preprocessed video inputs are depicted in Figure 3.

The subsequent validation of the NVI model demonstrated its potential in nonverbal behaviour analysis, approximating the accuracy of individual human raters. The ICC value that represents the correlation among the human ratings of the overall NVI of all three human raters and the NVI algorithm using the validation data set was .690. For further information about the NVI model and its subcomponents, see Petković et al. (2024).

Having confirmed the model's ability to accurately assess teachers' NVI, it was then applied to analyse an additional 220 30‐s segments from the TALIS video corpus. Consequently, NVI data from a total 623 video segments were used for the subsequent path analyses.

#### Survey measures

##### Teacher self‐efficacy for student engagement (Time 1)

The four‐point Likert scale ranged from 1 (*not at all*) to 4 (*very much*) and assessed class‐specific teacher self‐efficacy for student engagement in line with Tschannen‐Moran and Woolfolk Hoy (2001). The scale instruction was ‘How much can you do the following in your class?’ followed by four items. An example item is ‘Convincing the students in this class that they can achieve good performance in school.’ Reliability was acceptable, *ω* = .73.

##### Teachers' enthusiastic teaching behaviours during the lesson (assessed after the lesson; Time 3)

The four‐point Likert scale ranged from 1 (*never or almost never*) to 4 (*always or almost always*) and assessed students' perceptions of enthusiastic teaching behaviours in class during the lesson in regard to the topic of the lesson. The scale instruction was ‘How would you describe your teacher during the unit on quadratic equations?’ followed by eight items. Example items are ‘Our mathematics teacher was totally involved in the topic of quadratic equations’ and ‘Our mathematics teacher expressed that the topic of <quadratic equations> is important to him/her’. Reliability at Time 2 was good, *ω*_within_ = .86, *ω*_between_ = .98.

##### Students' mathematics interest (Time 1, Time 3, Time 4)

The four‐point Likert scale ranged from 1 (*strongly disagree*) to 4 (*strongly agree*) and assessed at Time 1 (pretest before the lesson) students' personal interest in mathematics classes with the current teacher using the scale instruction ‘How do you think about mathematics?’ followed by three items. An example item is ‘I often find what we cover in math class to be really exciting’. At Time 2 (posttest directly after the lesson), the items referred to students' interest in the content of the [quadratic equations] lesson. An example item is ‘I was interested in the topic of <quadratic equations>’. At Time 3 (follow‐up; 6 weeks after the lesson), items again addressed students' general interest in mathematics classes with the current teacher (e.g. ‘I often find what we cover in math class to be really exciting’). The scale reliabilities were good, see Table 1 (L2) and Table S2 (L1).

**TABLE 1:** Descriptive statistics and manifest correlations at the classroom level (L2).

	1	2	3	4	5	6	7
(1) Teacher SE T1							
(2) NVI T2	.26						
(3) Enthusiastic behaviours T3	.37***	.34***					
(4) Student interest T1	.41***	.21^c^	.58***				
(5) Student interest T3	.25^c^	.33***	.59***	.67***			
(6) Student interest T4	.31*	.47***	.47***	.80***	.66***		
(7) Teacher experience	.19	−.05	.02	−.01	.18	.20	
*M*	2.79	.55	2.99	2.40	2.17	2.30	1.94
SD	.42	.05	.30	.29	.27	.24	1.04
Min.	1.00	.44	1.00	1.00	1.00	1.00	1.00
Max.	4.00	.71	4.00	4.00	4.00	4.00	4.00
ICC(1)	–	–	.22	.11	.10	.10	–
*ω*_between_	–	–	.98	.98	.97	.86	–

*Note*: T1 = Time 1; T2 = Time 2; T3 = Time 3. Correlations are calculated at L2.

Abbreviations: ICC, intraclass correlation coefficient; NVI, nonverbal immediacy score; Teacher SE, teacher self‐efficacy.

^c^ *p* < .10, *p < .05, ***p < .001.

##### Covariates

Teachers were asked ‘How many years of work experience do you have as a mathematics teacher regardless of whether you worked full‐time or part‐time?’ and the response categories were coded as in the TALIS data set, ranging from 1 (1–10 years) to 4 (31–49 years).

### Statistical analyses

Intraclass correlation coefficients, reported in Table 1. ICC(1) values indicate how much variance is located at the highest level of analyses (L2) in multilevel analyses, in our case, at the classroom level. Values greater than .05 are considered sufficient for using the multilevel approach (Lüdtke et al., 2008). In our study, 10% of the variance in students' interest at each time point was attributable to students' membership in a specific classroom. A total of 22% of student‐perceived teachers' enthusiastic teaching behaviours during the lesson, assessed after the lesson, were explained by students' membership in the same classroom. Given the substantial amount of variance at L2, we applied a multilevel modelling approach with students (Level 1) nested in classrooms (Level 2).

Prior to the hypothesis testing, we tested metric invariance for student interest across time and across levels of analysis (L1, L2) following the steps described by Byrne (2004) and the cut‐off criteria for measuring non‐invariance in line with Chen (2007). The results of the invariance testing are reported in Table S.1 in the supplemental material. Results showed the metric invariance of student interest a cross time and levels.

Simulation studies on adequate sample sizes in multilevel modelling have led to rules of thumb that help guide choices about necessary sample sizes. For multilevel modelling, a sample size of 30 groups at L2 has been proposed as a minimum requirement to achieve unbiased standard errors (Maas & Hox, 2005), which is important because biased standard errors may lead to overestimates or underestimates of statistical power. In our study, given the expected effect sizes, the sample at L2 is likely at the lower bound for ensuring a sensitive test of the hypothesised relationships. For this reason, we applied manifest multilevel modelling (Marsh et al., 2009), a modelling procedure that makes efficient use of the available data. The procedure has been shown to result into lower standard errors while avoiding biased estimation of the model parameters in question. We tested our hypotheses with two models. Model 1 was a less restrictive model and included all relevant direct and indirect regression path between all study variables. Model 2 was a more restrictive model and included only the sequence of effects depicted in Figure 1. Both models included students' interest at Times 1, 3 and 4 at the student level (L1). At the classroom level, the models included teachers' self‐efficacy (Time 1), teachers' work experience (Time 1), students' interest (Times 1, 3 and 4), teachers' nonverbal immediacy (NVI) (Time 2) and student‐reported teachers' enthusiastic teaching behaviours (Time 3). Student interest (Time 1) was group‐mean centred, meaning that the mean of classroom j is subtracted from the score of student i in classroom j (Marsh et al., 2009). Indirect effects defined as products of regression coefficients were tested with 95% confidence intervals (CIs).

Mplus Version 8.3 was used for all analyses (Muthén & Muthén, 1998–2019), and maximum likelihood estimation with robust standard errors was applied. Goodness of model fit was evaluated using the following criteria: the comparative fit index (CFI), root mean square of approximation (RMSEA) and standardized root mean residual (SRMR_within_, _between_). As a rule of thumb, CFI values greater than or equal to .95 as well as RMSEA values below or equal to .06 and SRMRwithin/between values below or equal to .10 (Hu & Bentler, 1999) indicate highly satisfactory levels of model fit. However, researchers should not overly rely on the golden rules of fit (Hsu et al., 2015; Marsh et al., 2004), and particularly the SRMR value has been shown to be sensitive for sample size (Marsh et al., 2004). Missing data were handled by means of full‐information maximum likelihood estimation (FIML).

## RESULTS

### Descriptives

Descriptive statistics for all constructs at the class level are reported in Table 1. Descriptive statistics for Level 1 constructs are reported in Table S2. TSESE before the lesson (Time 1) was positively but marginally significantly (*p* < .10) associated with teachers' NVI during the lesson (Time 2). Teachers' NVI (Time 2) was positively and statistically significantly associated with aggregated student‐reported teachers' enthusiastic teaching behaviours during the lesson and with students' interest at Times 1 and 3 at the class level, but only marginally significantly and positively with students' interest at Time 4. Student‐reported class‐level teachers' enthusiastic teaching behaviours during the lesson was positively and significantly associated with students' interest at each time point.

### TSESE, NVI, enthusiastic teaching and student interest

The model fit of Model 1 that included all direct and indirect paths was acceptable: *χ* ^2^ = 681.15, df = 279; CFI = .93, TLI = .92, RMSEA = .04, SRMRwithin = .04, SRMRbetween = .13. Coefficients are depicted in Table 2. TSESE before the lesson (Time 1) was positively and significantly related to teachers' NVI during the lesson (Time 2) and to class‐level enthusiastic teaching behaviours, assessed after the lesson (Time 3). Teachers' NVI during the lesson (Time 2) was positively related to class‐level enthusiastic teaching behaviours, reported after the lesson (Time 3). Neither TSESE before the lesson (Time 1) nor NVI during the lesson (Time 2) or student‐reported enthusiastic teaching behaviours (Time 3) were significantly related to students' interest after the lesson (Time 3), or 6 weeks later (Time 4), when controlling for interest at Time 1. Indirect effects were not significant in this model.

**TABLE 2:** Model 1: Standardized coefficients of the latent‐manifest model.

Variable L1	Student int. T3				Student int. T4			
	*β*	SE	*p*	95% CI	*β*	SE	*p*	95% CI
Student int. T1	.60	.03	.001	.535, .661	.43	.05	.001	.336, .530
Student int. T3					.47	.04	.001	.386, .561
L1: *R* ^2^	.36***	.66***						

Variable L2	NVI T2				Enthu T3				Student int. T3				Student int. T4			
	*β*	SE	*p*	95% CI	*β*	SE	*p*	95% CI	*β*	SE	*p*	95% CI	*β*	SE	*p*	95% CI
TSESE T1	.38	.18	.034	.029, .738	.28	.13	.029	.113, .721	−.31	.20	.119	−.704, .080	−.27	.38	.475	−1.022, .476
NVI T2					.42	.16	.007	.028, .522	.22	.14	.119	−.057, .503	−.04	.21	.844	−.453, .371
Enthu T3									.26	.14	.070	−.021, .531	.07	.23	.771	−.378, .510
Student int. T1									.75	.12	.001	.524, .975	1.32	.39	.001	.554, 2.076
Student int. T3													−.44	.38	.249	−1.187, .308
Teacher exp	−.18	.16	.273	−.488, .138	−.05	.20	.797	−.450, .346	.29	.12	.020	.045, .526	.37	.31	.231	−.232, .964
*R* ^2^	.15				.32*				.64***				.91***			

*Note*: The correlation between TSESE and teacher exp. was *φ* = .23, SE = .16, *p* = .170, and between TSESE and students' interest T1 was *φ* = .58, SE = .14, *p* = .001.

Abbreviations: Enthu, student‐reported teacher enthusiastic teaching; NVI, teachers' nonverbal immediacy; Student int., students' interest; Teacher exp., teachers' teaching experience in years; TSESE, teachers' self‐efficacy for student engagement.

*p < .05, ***p < .001.

The model fit of Model 2 that only included the sequential effects depicted in Figure 1 was acceptable: *χ* ^2^ = 707.26, df = 290; CFI = .93, TLI = .92, RMSEA = .04, SRMRwithin = .04, SRMRbetween = .18. Coefficients are depicted in Table 3. TSESE before the lesson (Time 1) was positively and significantly related to teachers' NVI during the lesson (Time 2). Teachers' NVI during the lesson (Time 2) was positively related to class‐level enthusiastic teaching behaviours, reported after the lesson (Time 3). Student‐reported enthusiastic teaching behaviours, assessed after the lesson (Time 3) was significantly and positively associated with students' interest, assessed after the lesson (Time 3) when controlling for students' prior interest before the lesson (Time 1). Students' interest after the lesson (Time 3) was significantly and positively related to students' interest 6 weeks later (Time 4). Given the modelling procedure used, adjusting for students' previous interest means estimating the additional effect of other predictors for students representing an average interest level in their respective classroom at the adjusted measurement time point. As interest at Time 1 was not controlled, interest Time 3 predicted the level in students' interest at Time 4, but not the change in interest from Time 1 to Time 4. The indirect effect from TSESE to student‐rated teachers' enthusiastic teaching behaviours was not significant, *β*_ind_ = .18, SE = .05, *p* = .13 [95% CI −.05, .42]. The indirect effects from teachers' NVI (Time 2) to students' interest (Time 3) via student‐reported enthusiastic teaching behaviours (Time 3) (*β*_ind_ = .13, SE = .05, *p* = .01 [95% CI .03, .23]) and from student‐reported enthusiastic teaching behaviours (Time 3) to students' interest (Time 4) via students' interest (Time 3) (*β*_ind_ = .14, SE = .06, *p* = .03 [95% CI .01, .26]) were significant and positive.

**TABLE 3:** Model 2: Standardized coefficients of the latent‐manifest model.

Variable L1	Student int. T3				Student int. T4			
	*β*	SE	*p*	95% CI	*β*	SE	*p*	95% CI
Student int. T1	.60	.03	.001	.535, .660	.43	.05	.001	.336, .529
Student int. T3					.47	.04	.001	.384, .560
L1: *R* ^2^	.36***				.65***			

Variable L2	NVI T2				Enthu T3				Student int. T3				Student int. T4			
	*β*	SE	*p*	95% CI	*β*	SE	*p*	95% CI	*β*	SE	*p*	95% CI	*β*	SE	*p*	95% CI
TSESE T1	.40	.20	.048	.003, .793												
NVI T2					.45	.11	.001	.235, .672								
Enthu T3									.29	.12	.021	.043, .529				
Student int. T1									.67	.10	.001	.464, .869				
Student int. T3													.47	.15	.002	.179, .767
Teacher exp																
*R* ^2^	.16				.20*				.56***				.22			

*Note*: The correlation between TSESE and teacher exp. was *φ* = .19, SE = .16, *p* = .220, and between TSESE and students' interest T1 was *φ* = .50, SE = .13, *p* = .001. In this model, we correlated teacher exp. with NVI and Enthu, but both coefficients were not significant (NVI: *φ* = −.15, SE = .16, *p* = .346, Enthu: *φ* = .04, SE = .20, *p* = .828). The residual of an indicator of the latent factor ‘student interest Time 3’ was originally estimated to values below zero, but not significantly different from zero. We fixed this residual to zero following Hox (2002, p. 215).

Abbreviations: Enthu, student‐reported teacher enthusiastic teaching; NVI, teachers' nonverbal immediacy; Student int., students' interest; Teacher exp., teachers' teaching experience in in years; TSESE, teachers' self‐efficacy for student engagement.

*p < .05, ***p < .001.

[Correction added on 5 February 2025, after the first online publication: Table 3 has been updated in this version.]

Our analyses draw on two (minimum) or three (maximum) lessons of the participating teachers, depending on the number of lessons they videorecorded for the TALIS study. However, because a review of the time scheduling of the videorecorded lessons showed that the third lesson was videorecorded in some cases after the posttest, we provide additional analyses of our models based on only the first two videorecorded lessons in Tables S.3 and S.4 in the supplemental material to ensure a meaningful causal order of effects. These additional analyses can be seen as a robustness check and reveal that machine‐learning classified NVI was in Model 1 and 2 positively and significantly related to student‐perceived enthusiastic teaching behaviours. Teachers' self‐efficacy did only in Model 2 significantly and positively relate to machine‐learning classified NVI when only including two lessons.

## DISCUSSION

Addressing the paucity of research examining processes that underlie the effects from teacher to student motivation, this study aimed to uncover the links through which teachers' self‐efficacy for student engagement affects their instructional behaviours, which then ultimately relate to student interest. Our key findings revealed that teachers' NVI, objectively assessed via machine learning, was significantly and positively related to the students' ratings of enthusiastic teaching behaviours. Teachers' self‐efficacy for student engagement (TSESE) was related to their NVI across the tested models. However, indirect effects from TSESE to student‐rated enthusiastic teaching via NVI was not significant. Our study was innovative, as we assessed teachers' NVI via objective situation‐specific measures and applied a new theory‐guided AI‐based assessment approach using computational modelling based on classroom video data.

### TSESE, teachers' behaviours, and student interest: Is NVI the missing link?

In line with our assumptions (H1), we found that teachers who reported before the lesson that they believed themselves to be capable of motivating the students in their class expressed high levels of nonverbal immediacy. In turn, teachers with low levels of self‐efficacy for student engagement in a target class displayed low levels of NVI during the lesson. It is important to note that the direct effect of TSESE on teachers' NVI was significant across the tested models, However, probably due to the small sample size, the indirect effects from TSESE to student‐rated enthusiastic teaching via NVI was not significant with *p* = .052. Thus, there was a sequence of effects rather than an indirect pathway. An explanation might be the relatively small sample size at the classroom level of 46 teachers, which led to increased standard errors in our second less restrictive model. Our findings need to be replicated with larger samples particularly at the classroom level in future studies.

In line with our assumptions (H2) and following the theoretical assumptions of approaches studying nonverbal immediacy (Mehrabian, 1968; Richmond et al., 2003), we found that teachers' NVI during the lesson positively and significantly related to students' perceived enthusiastic teaching behaviours during the lesson, as reported by students after the lesson. Our findings can be seen as a validation of the machine‐learning assessed NVI as the results of the computational model of NVI corresponded with students' ratings of teachers' enthusiastic teaching behaviours, and thus, with a conceptually closed theoretical construct. Methodologically, our findings point to machine learning as an option to classify teaching behaviours for research in a resource‐efficient way. More research with different data sets and different teaching situations is needed to validate this approach further. However, we believe that classroom observation studies could largely benefit from our approach. On a theoretical level, we were able to show a sequence from TSESE to NVI, and from NVI to teaching behaviours. Thus, nonverbal behaviours of the teacher in class might be an explanation for why motivated teachers provide high‐quality instruction in class. Prior work has pointed out a deficiency of research of the processes linking teacher motivation to actual teaching behaviours (Lazarides et al., 2024). Our study partially closed this knowledge gap by revealing that teachers' nonverbal behaviours during the interactions with their students might be a part of the missing link.

In line with our expectations (H3) and work on teacher enthusiastic teaching behaviours (Frenzel et al., 2018), we further found that student reports of their teachers' enthusiastic teaching behaviours during the lesson (assessed directly after the lesson) was positively associated with students' domain‐specific interest directly after the lesson. However, Model 1 shows that neither TSESE nor NVI or enthusiastic teaching behaviours had a longitudinal effect on students' interest 6 weeks after the lesson (Time 4) when controlling for students' baseline interest (Time 1). One explanation for this finding might be the high stability of students' interest across time. The results of Model 2 show that when baseline interest was not controlled for, student‐reported enthusiastic teaching behaviours (Time 3) were positively related to students' interest 6 weeks after the lesson (Time 4) via students' interest directly after the lesson (Time 3). However, because both, student‐reported enthusiastic teaching behaviours and students' interest (Time 3) were assessed at the same time point, the directionality of effects cannot be disentangled in our models.

### Implications for research and educational practice

Our findings show that nonverbal communication in terms of body language, facial expressions and physical proximity foster student perceptions of enthusiastic teaching behaviours and subsequent student interest. Thus, teacher education and training that includes a focus on NVI might benefit both teaching and learning. Instructional strategies for teachers should be highlighted that allow them to express enthusiasm and interact closely with students, thereby creating a more engaging and dynamic learning experience. The present study showed that TSESE was prospectively related to teacher NVI; however, fostering teachers' NVI could potentially also shape their TSESE, which is a question left open for future research. Another question for future work would be in what terms the lesson‐specific interactions in our study are embedded in more long‐term, dynamic effects among teachers and their students. Thus, multi‐wave studies that comprise multiple lessons embedded in multiple measurement occasions assessed over longer time spans are needed to examine how much the relations between teachers' nonverbal behaviours and their students' perceptions thereof are across lessons and across longer time spans.

### Strengths and limitations

The longitudinal and multimodal design of our study is a definite strength, as it allows us to examine how the change in mean values of students' group‐level interest across time, from before the lesson to 6 weeks after the lesson, depends on mediational processes that transmit teachers' self‐reported self‐efficacy through their objectively assessed behaviours in class, and students' perceptions of these behaviours. However, our study also has limitations. First, the sample was limited to 50 teachers and their classrooms, and although this sample size is sufficient to test our hypotheses with considerable statistical power, the complex multilevel model that we tested should be validated in larger samples. Second, our findings are limited to the domain of mathematics, and more specifically to lessons on quadratic equations, and teachers' NVI behaviours might differ depending on the subject they teach (Goetz et al., 2013). Therefore, further research is needed to validate the NVI algorithm by applying it to various mathematics topics and subject domains. Third, the different levels of specificity in the measures are a unique feature of the data, enabling us to examine whether and to what extent teachers shape students' learning in particular classroom moments, which may, in turn, lead to more global manifestations in students' motivation. We sincerely believe that this operationalization effectively reflects teacher effects on learning processes and their potential consequences for students. It could be argued that variations in the level of specificity in the measures may explain weak relationships between constructs. For instance, the relationship between student‐rated enthusiastic teaching behaviours (lesson‐specific, topic‐related) and mathematics interest (general, not lesson‐specific; domain‐related) might be stronger if both measures referred to the same reference frame in terms of the teaching situation (lesson‐specific vs. general) and content area (topic vs. domain).

## CONCLUSIONS

Our study helped to shed light on the effects from TSESE to student‐perceived teaching behaviours and domain‐related interest by using a rigorous study design and an automated AI‐based assessment of teachers' NVI. Motivational research and classroom research are both currently developing towards situated approaches that include multimodal assessments of motivations and behaviours in instructional situations. Bringing together these fields and using new and innovative AI‐based methods would be promising for the future, enabling researchers to better understand how and why certain instructional settings decisively enhance students' motivation in class.

## AUTHOR CONTRIBUTIONS

**Rebecca Lazarides:** Conceptualization; investigation; funding acquisition; writing – original draft; methodology; visualization; writing – review and editing; formal analysis; project administration; resources. **Jonas Frenkel:** Data curation; writing – review and editing; project administration; visualization; methodology; validation. **Uroš Petković:** Methodology; visualization; project administration; data curation; validation. **Richard Göllner:** Writing – review and editing; formal analysis; supervision; methodology. **Olaf Hellwich:** Project administration; funding acquisition; investigation; methodology; software; supervision; resources.

## CONFLICT OF INTEREST STATEMENT

The authors declare no conflicts of interest to disclose.

## Supporting information




Data S1.

## Data Availability

The data that support the findings of this study are available on request from the corresponding author. The data are not publicly available due to privacy or ethical restrictions.

## Footnotes

^1^
We report here the weighted correlation coefficients from the meta‐analyses.

^2^
Three of the total 403 segments were excluded from NVI scoring by the human raters because the video quality did not allow an analysis of the full 30‐second duration. However, since analysis was still possible for the majority of the individual frames without restriction, these segments were retained for use in the respective submodels.
