# Supplementary material for: ‘No words’—Machine‐learning classified nonverbal immediacy and its role in connecting teacher self‐efficacy with perceived teaching and student interest
Source: Br J Educ Psychol. 2024 Dec 29;95(Suppl 1):S15–31. doi: 10.1111/bjep.12732 (PMC12427166; doi:10.1111/bjep.12732)
Supplement: Supplementary file 1 — Data S1. [file BJEP-95-S15-s001.docx]

**Supplemental Material**

**Table S.1**

*Student Models: Model Fit Indices for Measurement Invariance Testing of Student Interest Across Levels and Time*

|  | χ² | *df* | CFI | ΔCFI | RMSEA | ΔRMSEA | SRMR_within_ | ΔSRMR_w_ | SRMR_between_ | ΔSRMR_b_ |
| --- | --- | --- | --- | --- | --- | --- | --- | --- | --- | --- |
| Student Interest |  |  |  |  |  |  |  |  |  |  |
| Configural invar | 265.79 | 48 | .956 |  | 0.063 |  | 0.039 |  | 0.095 |  |
| Metric invar | 281.78 | 54 | .954 | -0.002 | 0.061 | -0.002 | 0.040 | 0.001 | 0.165 | 0.070 |

*Note*. *n* = 1,140. Models were evaluated in line with Chen (2007): changes of ≥ -.010 in CFI, supplemented by a change of ≥ .015 in RMSEA, and ≥ .030 in SRMR indicate non-invariance.

**Table S.2**

*Descriptive Statistics and Manifest Correlations at Level 1 (Student Level)*

|  | 1 | | | 2 | | 3 | | |
| --- | --- | --- | --- | --- | --- | --- | --- | --- |
| 1) Student interest T1 | |  |  | |  | |  |  |
| 2) Student interest T3 | | .58*** |  | |  | |  |  |
| 3) Student interest T4 | | .65*** | .59*** | |  | |  |  |
| *M* | | 2.40 | 2.16 | | 2.28 | | |  |
| *SD* | | 0.77 | 0.73 | | 0.67 | | |  |
| *Min.-*  *Max.* | | 1.00  4.00 | 1.00  4.00 | | 1.00  4.00 | | |  |
| *ω*within | | .852 | .812 | | .811 | | |  |

*Note.* T1 = Time 1; T2 = Time 2; T3 = Time 3*.* ^C^*p* < .10, **p* < .05, ***p* < .01, *** *p* < .001.

**Table S.3**

*Model 1: Standardized Coefficients of the Latent-Manifest Model with Two Videos*

| Variable L1 Student int T3 Student int T4 | | | | | | | | | | | | | | | | | | | | | | | | | | | | | | | | | | | | | | | | | | | |  |  |  |  |  |  |  |  |  |  |  |  |  |  |  |  |  |  |  |  |  |  |  |  |  |  |  |  |  |  |  |  |
| --- | --- | --- | --- | --- | --- | --- | --- | --- | --- | --- | --- | --- | --- | --- | --- | --- | --- | --- | --- | --- | --- | --- | --- | --- | --- | --- | --- | --- | --- | --- | --- | --- | --- | --- | --- | --- | --- | --- | --- | --- | --- | --- | --- | --- | --- | --- | --- | --- | --- | --- | --- | --- | --- | --- | --- | --- | --- | --- | --- | --- | --- | --- | --- | --- | --- | --- | --- | --- | --- | --- | --- | --- | --- | --- | --- |
|  | β | | | | | | | *SE* | | | | *p* | | 95% CI | | | | β | | | | *SE* | | | | | | *p* | | | | | | | | 95% CI | | | | | | |  |  |  |  |  |  |  |  |  |  |  |  |  |  |  |  |  |  |  |  |  |  |  |  |  |  |  |  |  |  |  |  |  |
| Student int T1 | .60 | | | .03 | | | | | .001 | | | | [.535, .661] | | | | | | .43 | | | | | .05 | | | | | .001 | | | | | | | | [.336, .530] | | | | | | | | |  |  |  |  |  |  |  |  |  |  |  |  |  |  |  |  |  |  |  |  |  |  |  |  |  |  |  |  |  |  |
| Student int T3 |  | | |  | | | | |  | | | |  | | | | | | .47 | | | | | .04 | | | | | .001 [.386, .561] | | | | | | | | | | | | | | | | | | | | | | | |  | | | | | |  |  |  |  |  |  |  |  |  |  |  |  |  |  |  |  |  |
| L1: *R*² | .36*** | | | | | | | | .66*** | | | | | | | | | | | | | | | | | | | | | | | | | | | | | | | | | | | | | | |  |  |  |  |  |  |  |  |  |  |  |  |  |  |  |  |  |  |  |  |  |  |  |  |  |  |  |  |
| NVI T2 | | | | | | | | | | | | | | | | | | | Enthu T3 | | | | | | | | | | | | | | | | | | | | | | Student int T3 | | | | | | | | | | | | | | | | | | Student int T4 | | | | | | | | | | | |  |  |  |  |  |
| Variable L2 | β | | | *SE* | | | | | *p* | | | | 95% CI | | | | | | | β | | | | | | *SE* *p*  95% CI | | | | | | | | | | | | | | | | | | β | | | | *SE* | | | | *p* | | 95% CI | | | | | | | | | | β | | | | *SE* | | *p* | 95% CI |  |  |  |  |
| TSESE T1 | .35 | | | .29 | | | | | .231 | | | | [-.225, .931] | | | | | | .44 | | | | | .15 | | | | | .003 | | | | | [.145, .736] | | | | | | | | | | -.30 | | | | .21 | | | | .152 | | | | | [-.708, .110] | | | | -.26 | | | | | | .38 | | .484 | | [-1.001, .475] | | | |  |
| NVI T2 |  | | |  | | | | |  | | | |  | | | | | | .24 | | | | | .11 | | | | | .031 | | | | | [.022, .453] | | | | | | | | | | .20 | | | | .18 | | | | .258 | | | | | [-.146, .544] | | | | -.08 | | | | | | .23 | | .741 | | [-.528, .376] | | | |  |
| Enthu T3 |  | | |  | | | | |  | | | |  | | | | | |  | | | | |  | | | | |  | | | | |  | | | | |  | | | | | .28 | | | | .14 | | | | .056 | | | | | [-.007, .557] | | | | .07 | | | | .22 | | | | .770 [-.370, .499] | | | | | |  |
| Student int T1 |  | | | |  | | | |  | | | |  | | | | |  | | | | |  | | | |  | | | | |  | | | | | |  | | | | | | .74 | | | .11 | | | .001 | | | | | [.507, .972] | | | | | 1.30 | | | | | .39 | | | | .001 | | [.545, 2.055] | | | | |
| Student int T3 | |  | | | |  | | | |  | | | | | |  | |  | | |  | | | | | | | | | |  | | | |  | | | | |  | | | | |  | | | | | |  | | | | | | |  | | | | -.41 | | | .39 | | | | .288 | | [-1.172, .348] | | |  |  |
| Teacher exp | | | -.19 | | | | .16 | | | | .230 | | | | [-.516, .124] | | -.06 | | | | | | | | .21 | | | | | .792 | | | [-.450, .359] | | | | | | | | | .29 | | | | | .13 | | .024 | | | | | | | [.038, .534] | | | | | | | .37 | | | .31 | | | .231 | | [-.246, .948] | | | | |
| *R*² .13 .31* .63*** .91*** | | | | | | | | | | | | | | | | | | | | | | | | | | | | | | | | | | | | | | | | | | | | | | | | | | | | | | | | | | | | | | | | | | | | | | | | |  |  |  |
| χ² (256) = 658.461, CFI = .93, TLI = .92, RMSEA = .037, SRMRwithin = .036, SRMRwithin = .133 | | | | | | | | | | | | | | | | | | | | | | | | | | | | | | | | | | | | | | | | | | | | | | | | | | | | | | | | | | | | | | | | | | | | | | | | |  |  |  |

*Note.* TSESE = teachers’ self-efficacy for student engagement. Student int = students’ interest. NVI = teachers’ nonverbal immediacy. Enthu = student-reported teacher enthusiastic teaching. Teacher exp = teachers’ teaching experience in in years.

**Table S.4**

*Model 2: Standardized Coefficients of the Latent-Manifest Model With Two Videos*

| Variable L1 Student int T3 Student int T4 | | | | | | | | | | | | | | | | | | | | | | | | | | | | | | | | | | | | | | | | | | | |  |  |  |  |  |  |  |  |  |  |  |  |  |  |  |  |  |  |  |  |  |  |  |  |  |  |  |  |  |  |  |  |
| --- | --- | --- | --- | --- | --- | --- | --- | --- | --- | --- | --- | --- | --- | --- | --- | --- | --- | --- | --- | --- | --- | --- | --- | --- | --- | --- | --- | --- | --- | --- | --- | --- | --- | --- | --- | --- | --- | --- | --- | --- | --- | --- | --- | --- | --- | --- | --- | --- | --- | --- | --- | --- | --- | --- | --- | --- | --- | --- | --- | --- | --- | --- | --- | --- | --- | --- | --- | --- | --- | --- | --- | --- | --- | --- | --- |
|  | β | | | | | | | *SE* | | | | *p* | | 95% CI | | | | β | | | | *SE* | | | | | | *p* | | | | | | | | 95% CI | | | | | | |  |  |  |  |  |  |  |  |  |  |  |  |  |  |  |  |  |  |  |  |  |  |  |  |  |  |  |  |  |  |  |  |  |
| Student int T1 | .60 | | | .03 | | | | | .001 | | | | [.535, .660] | | | | | | .43 | | | | | .05 | | | | | .001 | | | | | | | | [.336, .529] | | | | | | | | |  |  |  |  |  |  |  |  |  |  |  |  |  |  |  |  |  |  |  |  |  |  |  |  |  |  |  |  |  |  |
| Student int T3 |  | | |  | | | | |  | | | |  | | | | | | .47 | | | | | .04 | | | | | .001 [.384, .560] | | | | | | | | | | | | | | | | | | | | | | | |  | | | | | |  |  |  |  |  |  |  |  |  |  |  |  |  |  |  |  |  |
| L1: *R*² | .36*** | | | | | | | | .65*** | | | | | | | | | | | | | | | | | | | | | | | | | | | | | | | | | | | | | | |  |  |  |  |  |  |  |  |  |  |  |  |  |  |  |  |  |  |  |  |  |  |  |  |  |  |  |  |
| NVI T2 | | | | | | | | | | | | | | | | | | | Enthu T3 | | | | | | | | | | | | | | | | | | | | | | Student int T3 | | | | | | | | | | | | | | | | | | Student int T4 | | | | | | | | | | | |  |  |  |  |  |
| Variable L2 | β | | | *SE* | | | | | *p* | | | | 95% CI | | | | | | | β | | | | | | *SE* *p*  95% CI | | | | | | | | | | | | | | | | | | β | | | | *SE* | | | | *p* | | 95% CI | | | | | | | | | | β | | | | *SE* | | *p* | 95% CI |  |  |  |  |
| TSESE T1 | .46 | | | .22 | | | | | .041 | | | | [.019, .904] | | | | | |  | | | | |  | | | | |  | | | | |  | | | | | | | | | |  | | | |  | | | |  | | | | |  | | | |  | | | | | |  | |  | |  | | | |  |
| NVI T2 |  | | |  | | | | |  | | | |  | | | | | | .45 | | | | | .13 | | | | | .001 | | | | | [.194, .708] | | | | | | | | | |  | | | |  | | | |  | | | | |  | | | |  | | | | | |  | |  | |  | | | |  |
| Enthu T3 |  | | |  | | | | |  | | | |  | | | | | |  | | | | |  | | | | |  | | | | |  | | | | |  | | | | | .29 | | | | .12 | | | | .021 | | | | | [.043, .527] | | | |  | | | |  | | | |  | | | | | |  |
| Student int T1 |  | | | |  | | | |  | | | |  | | | | |  | | | | |  | | | |  | | | | |  | | | | | |  | | | | | | .67 | | | .10 | | | .001 | | | | | [.459, .870] | | | | | .47 | | | | | .15 | | | | .002 | | [.182, .766] | | | | |
| Student int T3 | |  | | | |  | | | |  | | | | | |  | |  | | |  | | | | | | | | | |  | | | |  | | | | |  | | | | |  | | | | | |  | | | | | | |  | | | |  | | |  | | | |  | |  | | |  |  |
| Teacher exp | | |  | | | |  | | | |  | | | |  | |  | | | | | | | |  | | | | |  | | |  | | | | | | | | |  | | | | |  | |  | | | | | | |  | | | | | | |  | | |  | | |  | |  | | | | |
| *R*² .21 .20 .56*** .26 | | | | | | | | | | | | | | | | | | | | | | | | | | | | | | | | | | | | | | | | | | | | | | | | | | | | | | | | | | | | | | | | | | | | | | | | |  |  |  |
| χ² (267) = 683.285, CFI = .93, TLI = .92, RMSEA = .037, SRMRwithin = .036, SRMRwithin = .183. | | | | | | | | | | | | | | | | | | | | | | | | | | | | | | | | | | | | | | | | | | | | | | | | | | | | | | | | | | | | | | | | | | | | | | | | |  |  |  |

*Note.* TSESE = teachers’ self-efficacy for student engagement. Student int = students’ interest. NVI = teachers’ nonverbal immediacy. Enthu = student-reported teacher enthusiastic teaching. Teacher exp = teachers’ teaching experience in in years. The residual of an indicator of the latent factor ‘student interest Time 3’ was originally estimated to values below zero, but not significantly different from zero. We fixed this residual to zero.
